# FOXM1 predicts disease progression in non-muscle invasive bladder cancer

**DOI:** 10.1007/s00432-018-2694-5

**Published:** 2018-06-29

**Authors:** Sebastien Rinaldetti, Ralph Wirtz, Thomas Stefan Worst, Arndt Hartmann, Johannes Breyer, Lars Dyrskjot, Philipp Erben

**Affiliations:** 1 Department of Hematology and Oncology, University Medical Centre Mannheim, Medical Faculty Mannheim, University of Heidelberg, Theodor-Kutzer-Ufer 1-3, 68167 Mannheim, Germany; 2 Stratifyer Molecular Pathology, Werthmannstraße 1, 50935 Cologne, Germany; 3 Department of Urology, University Medical Centre Mannheim, Medical Faculty Mannheim, University of Heidelberg, Theodor-Kutzer-Ufer 1-3, 68167 Mannheim, Germany; 4 Institute of Pathology, University Hospital Erlangen, Krankenhausstraße 8-10, 91054 Erlangen, Germany; 5 Department of Urology, University of Regensburg, Landshuter Str. 65, Regensburg, Germany; 6 Department of Molecular Medicine, Aarhus University Hospital, Aarhus, Denmark; 7 Department of Clinical Medicine, Aarhus University, Aarhus, Denmark

**Keywords:** Molecular subtypes, FOXM1, Progression-free survival, Biomarker, Bladder cancer, MKI67

## Abstract

**Purpose:**

The proto-oncogene forkhead box M1 (FOXM1) is associated with poor survival in many cancers. The impact of FOXM1 expression on progression-free survival (PFS) of non-muscle invasive bladder cancer (NMIBC) has not yet been investigated. The differential expression of FOXM1 between the different molecular NMIBC subtypes has further been assessed.

**Methods:**

Transcript levels of FOXM1 and MKI67 were determined in 460 NMIBC patients (UROMOL cohort) by RNA-Seq and validated in silico by the Chungbuk and Lund cohort (*n* = 277). FOXM1 and MKI67 cutoffs were identified by the minimal *p* value method. Variables were evaluated by multivariable Cox regression analyses in order to identify independent predictors.

**Results:**

FOXM1 is an independent predictor for PFS superior to current histological, clinical and molecular staging methods. Patients with high FOXM1 expression have a 6- to 8-fold higher risk of progression in multivariable analysis (*p* < 0.03). Highest transcript levels were found in the Class 2 and genomically unstable molecular NMIBC subtype (*p* < 0.03). The proto-oncogene further positively correlated with tumor grade and stage. NMIBCs with high FOXM1 expression showed a PFS advantage when treated with intravesical BCG instillation.

**Conclusion:**

FOXM1 is a highly prognostic marker for disease progression of NMIBC superior to current histological, clinical and molecular staging methods and MKI67. It is mainly expressed in the Class 2 and genomically unstable molecular bladder cancer subtypes. Its role in drug resistance development makes FOXM1 valuable biomarker for NMIBC risk stratification.

## Background

Non-muscle invasive tumors represent 75% of patients diagnosed with transitional cell carcinoma. Transurethral resection of bladder tumors (TURB), intravesical mitomycin C and bacillus Calmette–Guerin (BCG) instillation represent the current standard treatments for non-muscle invasive bladder cancer (NMIBC) (Babjuk et al. 2017). As 50–80% of pTa NMIBC have cancer recurrence and 10–30% of pT1 and CIS patients show disease progression, clinicopathologic parameters are insufficient for disease prediction (Prout et al. 1992; Ark et al. 2014). Given the biologic heterogeneity of bladder cancer, survival and progression varies even within the same stage. Thus molecular biomarkers are needed to improve prediction of treatment response or even for clinical decision-making in the sense of preemptive biomarkers (Youssef and Lotan 2011; Kluth et al. 2015). Based on recent data, the most promising results were provided by gene expression signatures of TURB samples and by liquid biopsies from urine or blood (Contreras-Sanz et al. 2017; Robertson et al. 2017). The elucidation of valuable invasive or non-invasive biomarkers or drug targets is still at its beginning.

The role of the proto-oncogene forkhead-box M1 (FOXM1) in carcinogenesis and drug resistance development is already well established and has been validated in many cancer types (Dai et al. 2015). FOXM1 originates from the forkhead gene family, was first identified in Drosophila and is characterized by a conserved 100-amino acid DNA-binding motif. It is involved as regulator in embryogenesis and numerous developmental processes (Ye et al. 1997). In adult organs, FOXM1 is mainly expressed in proliferating cells or induced by growth factor release. In this context, it is not surprising that FOXM1 serves as proto-oncogene in most cancers and aberrant expression or mutations constitute the origin of many treatment resistance mechanisms (Kwok et al. 2010; Kalin et al. 2011; Saba et al. 2016). Although FOXM1 is exclusively expressed in dividing cells, its targeting may result in many side effects given its involvement in angiogenesis, cell migration and epithelial–mesenchymal transition (Halasi and Gartel 2013). We have recently shown that FOXM1 is a predictor for overall and disease-specific survival in muscle invasive bladder cancer (MIBC) superior to the TNM staging system and MKI67 (Rinaldetti et al. 2017). A recent TCGA study further underlined the role of FOXM1 as regulator in MIBC (Robertson et al. 2017). As MKI67 is considered as the gold standard biomarker for proliferation and prognosis, the impact of FOXM1 needs yet again to be compared with the later (Rodríguez-Alonso et al. 2002).

Recent findings showed that bladder cancer can be subclassified in molecular subtypes with some similarities to breast cancer subtypes (Choi et al. 2014; Hedegaard et al. 2016; Robertson et al. 2017). These findings open the doors for personalized treatment concepts similar to those in breast cancer. That is why the subtype-specific expression of FOXM1 will be analyzed in this study. As the FOXM1 signaling network represents a valuable and promising target for further cancer treatment personalization, we here investigate its clinical impact in three cohorts with a total of 737 NMIBC patients.

## Methods

### Clinicopathologic characteristics

Patients and clinicopathologic data of 460 NMIBC patients from a European multicenter prospective study (UROMOL cohort) were investigated (Hedegaard et al. 2016). All samples have a carcinoma cell percentage > 50. Clinicopathologic characteristics are summarized in Tables 1 and 2. Sample collection procedures were published before (Hedegaard et al. 2016). All patients gave informed consent and the study was approved in all countries by institutional review boards or ethical committees. Expression data and clinicopathologic information from the Chungbuk (*n* = 104, GSE13507) and Lund (*n* = 173, GSE32894) cohort were used for validation (Kim et al. 2010; Sjödahl et al. 2012). All MIBC (T2–T4) patients were excluded.

**Table 1:** Clinicopathologic characteristics of the UROMOL cohort

UROMOL cohort: clinicopathologic characteristics (*n* = 460)	Median value (range) or absolute value (%)
Age	69 (23–96)
Male	357 (78%)
Grade	
High grade	176 (38%)
Low grade	277 (60%)
PUNLMP	7 (2%)
Stage	
CIS	3 (1%)
Ta	345 (75%)
T1	112 (24%)
Growth pattern	
Papillary	414 (97%)
Other (mixed, solid, unknown)	11 (3%)
BCG treatment	88 (20%)
FOXM1 expression	1.67 (0–5.7)
MKI67 expression	2.74 (0.26–6.24)
Follow-up duration (months)	33 (0–75)

**Table 2:** Clinicopathologic characteristics of the Chungbuk cohort

Chungbuk cohort: clinicopathologic characteristics (*n* = 104)	Median value (range) or absolute value (%)
Age	67 (24–88)
Male	87 (84%)
Grade	
High grade	18 (17%)
Low grade	86 (83%)
Stage	
Ta	24 (23%)
T1	80 (77%)
Intravesical therapy	56 (54%)
FOXM1 expression	8.08 (7.25–10.34)
MKI67 expression	7.62 (6.92–9.66)
Follow-up duration (months)	55 (2–137)

### Gene expression analyses

Gene expression analyses of FOXM1 and MKI67 are based on paired-end RNA-Seq (101 + 7 + 101 bp) analysis on an on an Illumina HiSeq 2000. Data were normalized as described before and log2 transformed. Cluster assignments were performed by ConsensusClusterPlus using the programming software R (version 3.2.2, R Foundation for Statistical Computing, Vienna, Austria) (Hedegaard et al. 2016).

Expression data of the Chungbuk and Lund cohort were based on Illumina human-6 v2.0 expression beadchip and Affymetrix Human Gene 1.0 ST Array analyses, respectively. Processed gene expression data, as used in the respective studies, were downloaded from the Gene Expression Omnibus database (Kim et al. 2010; Sjödahl et al. 2012).

### Statistical analyses

Cutoffs for FOXM1 and MKI67 were identified by the minimum p value method in order to stratify patients in a high- and low-risk group (Budczies et al. 2012). The hazard ratio of the cutoffs was estimated by multivariable Cox regression analysis. The Wald forward algorithm was used for testing significance. The stepwise entry criterion for covariates was *p* < 0.05 and the removal criterion consisted in *p* > 0.10. Association between variables was analyzed using Kruskal–Wallis test, Mann–Whitney *U* test or Spearman’s rank correlation. Kaplan–Meier estimates together with the log-rank test were used for survival analysis. In case the median survival rates could not be determined, they were specified as mean survival rates. The unadjusted significance level of 0.05 was considered for all statistical tests. Statistical analyses were performed using SPSS (version 20, IBM, Armonk, NY, USA).

## Results

### High correlation of FOXM1 with stage, grade and MKI67 expression

**Fig. 1:**
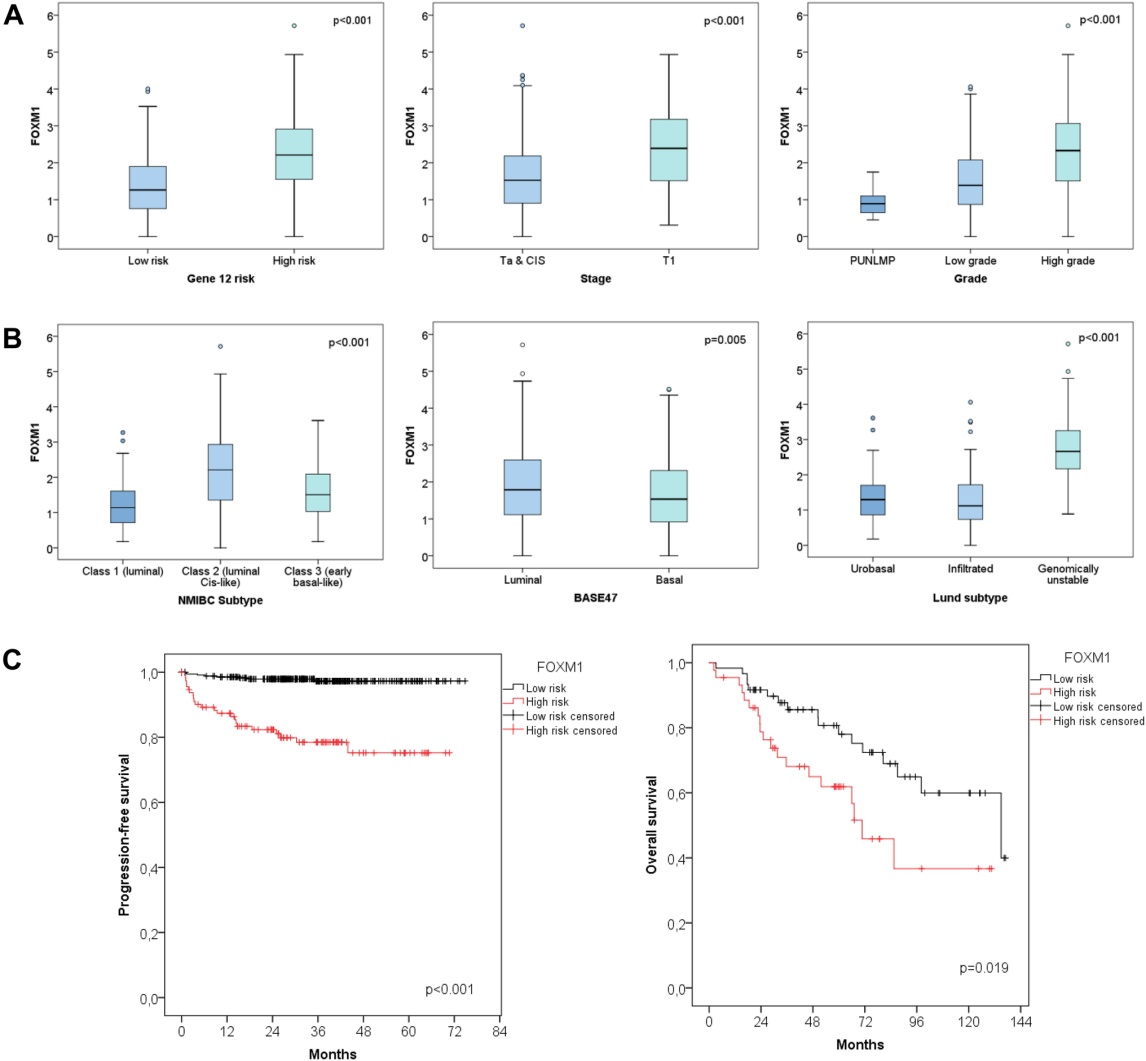
UROMOL cohort: **a** differential expression of FOXM1 based on tumor stage, grade and the 12-gene risk stratification. **b** Differential expression of FOXM1 based on the molecular bladder cancer subtype. **c** Kaplan–Meier plot for progression-free survival based on the FOXM1 risk stratification

**Fig. 2:**
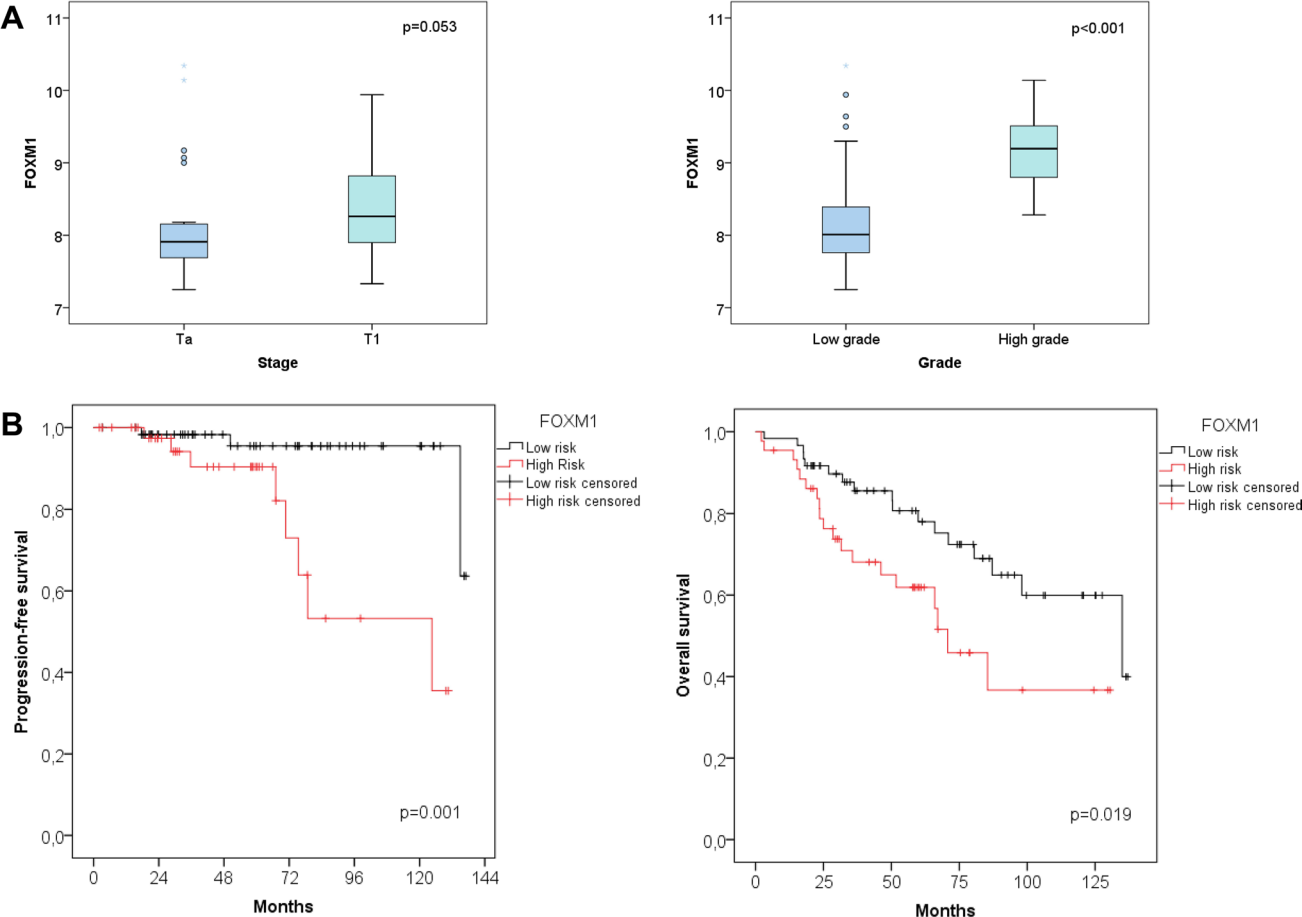
Chungbuk cohort: **a** differential expression of FOXM1 based on tumor stage and grade. **b** Kaplan–Meier plots of the Chungbuk cohort for progression-free survival and overall survival associated with the FOXM1 risk stratification

**Fig. 3:**
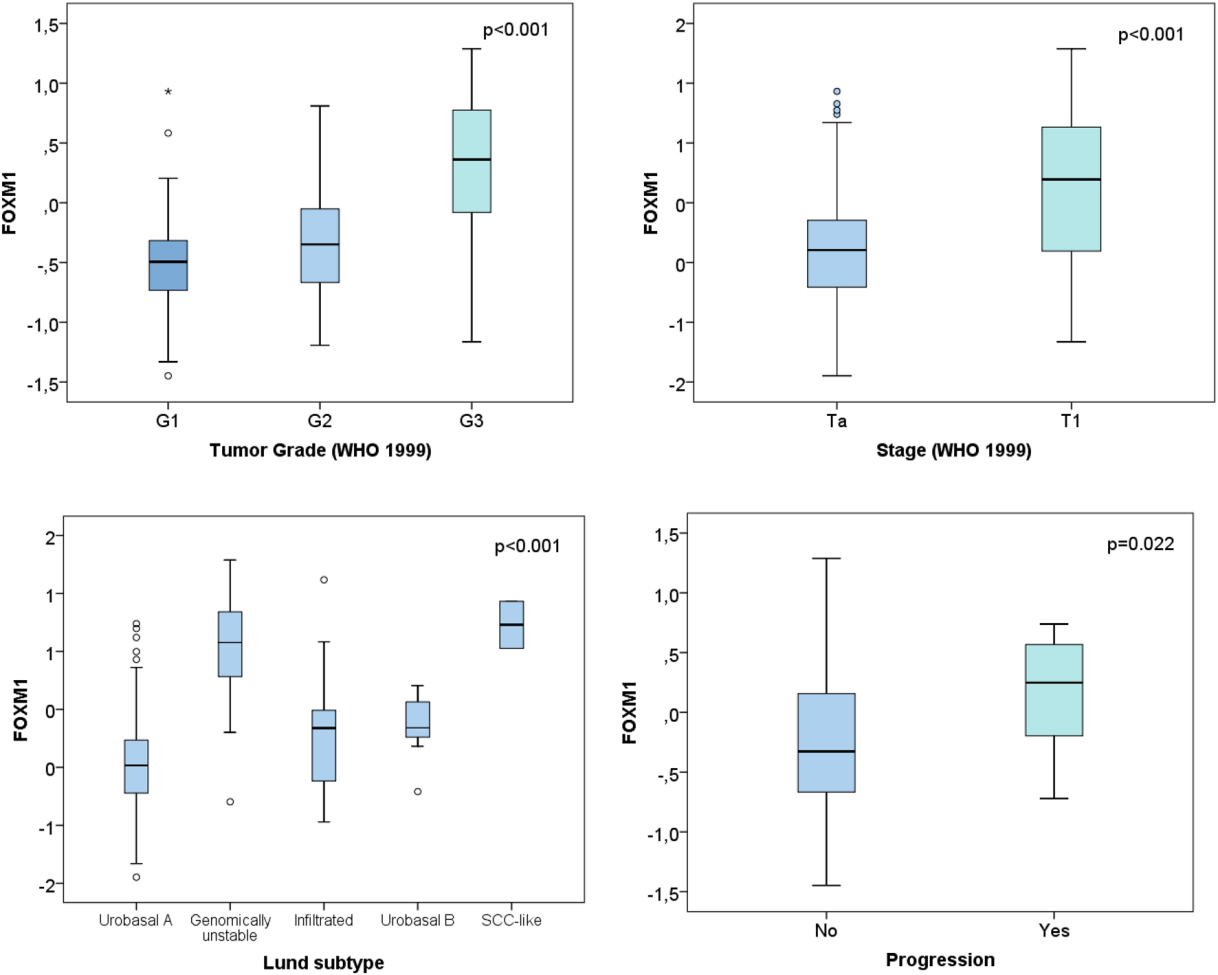
Lund cohort: correlation of FOXM1 with clinical and molecular characteristics of the Lund cohort

In both the UROMOL and Lund cohort FOXM1 was significantly higher in T1 vs Ta NMIBCs (Figs. 1a, 3, *p* < 0.001). Further, the expression correlated positively with tumor grade in the UROMOL, Chungbuk and Lund cohort (Figs. 1a, 2a, *p* < 0.001). The same tendency was observed for MKI67 (data not shown). This is in accordance with the high spearman correlation between FOXM1 and MKI67 of the UROMOL, Chungbuk and Lund cohorts (Spearman coefficient: 0.88 / 0.86 / 0.77, respectively, *p* < 0.001). The 12-gene risk score developed for predicting progression (Dyrskjøt et al. 2017) positively correlated with the FOXM1 expression (*p* < 0.001, Fig. 1a).

### FOXM1 is superior in predicting PFS than MKI67 and TNM

The cutoff levels for FOXM1 and MKI67 have been optimized for progression-free survival (PFS). For the Lund cohort no cutoff could be determined as duration data for PFS were not available. Poor PFS correlated with high FOXM1 or MKI67 expression. Cutoff values for risk stratification were calculated by the minimal *p* value method. A FOXM1 cutoff of 2.41 (median 1.66, 95% CI 1.72–1.91, Fig. 1c) and an MKI67 cutoff of 5.1 (median 1.67, 95% CI 1.72–1.91, figure not shown) have been determined for the UROMOL cohort. A FOXM1 cutoff of 8.27 (median 8.07, 95% CI 8.20–8.47) and a MKI67 cutoff of 8.04 (median 7.6, 95% CI 7.68–7.92, figure not shown) were chosen for the Chungbuk cohort (Fig. 2b, Kaplan–Meier plot for MKI67 not shown). In both the UROMOL and Chungbuk cohort, these FOXM1 and MKI67 cutoffs allowed a distinct risk stratification.

The molecular and clinicopathologic parameters of the UROMOL cohort, tested by univariable Cox regression analysis, are summarized in Table 3. The single variables retained by the multivariable Cox regression model as independent PFS predictors were age, stage and FOXM1 with a hazard ratio (HR) of 5.7 (95% CI 2.45–12.31, *p* < 0.001, Table 3). Indeed, the FOXM1 high-risk group showed a mean PFS of 57 months (*n* = 114, 95% CI 51.78–61.90) in contrast to the prolonged mean PFS of 73 months from the low-risk group (*n* = 346, 95% CI 72.08–74.35, *p* < 0.001, Fig. 1c).

**Table 3:** Uni- and multivariable analyses of the UROMOL cohort with progression-free survival as endpoint

Variables	Univariable Cox regression analyses			Multivariable Cox regression analyses		
	HR	95% CI	*p* value	HR	95% CI	*p* value
UROMOL cohort						
FOXM1 high vs low risk	9.8	4.40–22.03	< **0.001**	5.71	2.45–12.31	< **0.001**
MKI67 high vs low risk	10.6	4.07–27.72	< **0.001**			
Subtype (luminal cis-like vs rest)	5.5	1.21–8.30	**0.019**			
BASE47	0.88	0.43–1.78	0.717			
CIS signature	4.50	1.73–11.73	**0.002**			
Gene 12 risk signature	2.85	1.31–6.18	**0.008**			
Lund subtype (infiltrated vs rest)	0.14	0.04–0.46	**0.001**			
Stage (Ta, CIS vs T1)	9.22	4.24–20.05	< **0.001**	5.28	2.36–11.82	< **0.001**
Grade (low grade vs rest)	0.23	0.11–0.50	< **0.001**			
Age	1.06	1.02–1.10	0.005	1.04	1.00–1.08	**0.04**
Sex	0.82	0.37–1.83	0.630			
BCG treatment	0.57	0.20–1.64	0.300			

Bold values indicate statistical significiance (*p* < 0.05)

*HR* hazard ratio, *CI* confidence interval

The impact of FOXM1 has further been validated in silico by the independent Chungbuk cohort. The covariates age, grade, BCG instillation therapy, FOXM1 and MKI67 cutoff were included in the multivariable analysis (Table 4). However, the only covariate retained by the Cox regression model was the FOXM1 cutoff with an HR of 8.53 (95% CI 1.79–40.72, *p* = 0.007). The FOXM1 high-risk (*n* = 44) versus low-risk group (*n* = 60) showed a mean PFS of 98 months (95% CI 80.78–116.12) versus 132 months (95% CI 125.68–138, Fig. 2b). The same cutoff was tested for overall survival. The FOXM1 high-risk vs low-risk group (*n* = 60) showed a median OS of 70 months (95% CI 51.82–89.64) versus 135 months (95% CI 72.16–197.78, Fig. 2b).

**Table 4:** Uni- and multivariable analyses of the Chungbuk cohort with progression-free survival as endpoint

Variables	Univariable Cox regression analyses			Multivariable Cox regression analyses		
	HR	95% CI	*p* value	HR	95% CI	*p* value
Chungbuk cohort						
FOXM1 high vs low risk	8.53	1.79–40.72	**0.007**	8.53	1.79–40.72	**0.007**
MKI67 high vs low risk	6.07	1.69–21.80	**0.006**			
Intravesical therapy	3.61	0.94–13.83	0.061			
Stage (Ta vs T1)	1.41	0.35–5.64	0.625			
Grade (low vs high grade)	3.53	0.88–14.17	0.075			
Age	1.06	0.10–1.12	0.060			
Sex	27.78	0.02–3.24 × 10E4	0.356			

Bold values indicate statistical significiance (*p* < 0.05)

*HR* hazard ratio, *CI* confidence interval

### Aberrant expression of FOXM1 within molecular subtypes

The NMIBC subtypes developed by the UROMOL cohort were described recently by Hedegaard et al. They subclassified the NMIBC into three molecular subtypes: Class 1 (luminal), Class 2 (luminal CIS-like) and Class 3 (basal-like). FOXM1 was exclusively overexpressed in the Class 2 subtype (Fig. 1b, *p* < 0.001). The classification into luminal and basal based on the BASE47 signature from Damrauer et al. (2014) showed an overexpression of FOXM1 in the luminal subtype (Fig. 1b, *p* = 0.005). The molecular subtypes previously defined by Sjödahl et al. on the Lund cohort, included the subtypes described in Fig. 3. FOXM1 showed a distinct overexpression in the genomically unstable and SCC-like molecular subtype (*p* < 0.001). The NMIBC samples from the UROMOL cohort had additionally been clustered according to the main Lund subtypes: urobasal, infiltrated and genomically unstable. Also in the UROMOL cohort, the genomically unstable subtype showed highest FOXM1 transcript levels (Fig. 1b, *p* < 0.001).

### FOXM1 serves as prognostic marker for BCG instillation therapy

**Fig. 4:**
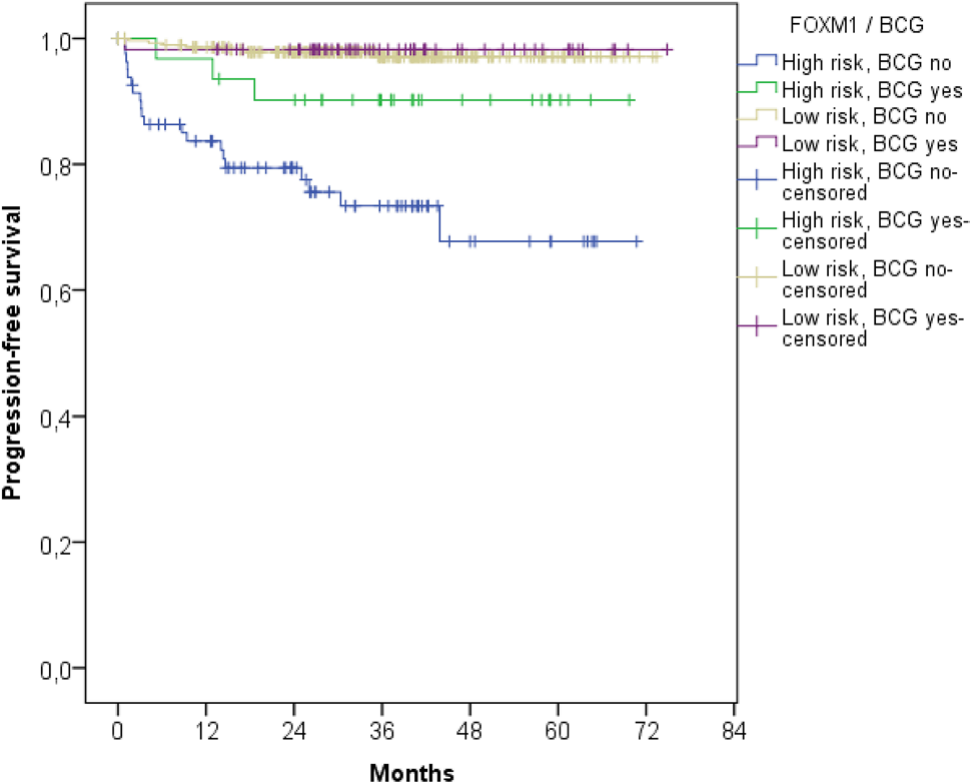
Kaplan–Meier plot of the UROMOL cohort for progression-free survival stratified by the FOXM1 expression and BCG treatment

Following the FOXM1 risk stratification, NMIBCs were further divided in patients with and without intravesical BCG instillation treatment (*p* < 0.001, Fig. 4). Patients with poorest PFS received no instillation treatment and had high FOXM1 expression. However, patients with FOXM1 overexpression benefited most from BCG instillation treatment and constitute an intermediate risk group. However, the BCG therapy had no impact on NMIBC patients with low FOXM1 transcript levels. The Kaplan–Meier estimates showed no differences in PFS in FOXM1 low-risk patients with and without BCG treatment (Fig. 4).

## Discussion

In this study, the prognostic and clinical impact of FOXM1 has been investigated retrospectively in a multicenter study and has been validated in published datasets. In order to evaluate its translational benefit, FOXM1 has been compared with MKI67 and relevant clinicopathologic parameters in multivariable analyses. Validation was performed by in silico data based on the Lund and Chungbuk cohort. FOXM1 showed a high correlation with MKI67, underlining its known role in cell proliferation and migration (Hamurcu et al. 2016). High FOXM1 transcript levels correlated with high tumor stage and grade. However, we managed to demonstrate that FOXM1 is an independent predictor for PFS superior to the TNM staging system and MKI67. Indeed, patients of the high-risk group characterized with high FOXM1 expression showed a 6- to 8-fold higher risk of progression (*p* < 0.001).

The UROMOL low-risk group comprised 75% of the patients, whereas in the Chungbuk cohort 58% of patients were included. This can be explained by the significantly higher portion of T1 NMIBCs of the Chungbuk cohort.

FOXM1 showed a distinct subtype-specific overexpression in the Class 2, genomically unstable and SCC-like subtypes (*p* < 0.001, Figs. 1b, 3). As published previously, the Class 2 subtype from Hedegaard at al. overlapped with the Lund subtypes ‘genomically unstable’ and ‘SCC-like’ (Hedegaard et al. 2016). FOXM1 is suspected to play a phenotype-determining role in the development of the molecular bladder cancer subtypes and promote its aggressiveness (Eriksson et al. 2015). The Class 2 subtype, characterized by an APOBEC-related mutational signature and the upregulation of the ERBB gene family, is known to have poorest PFS rate in NMIBCs (Hedegaard et al. 2016). Thus the overexpression of FOXM1 in this specific subtype seems plausible. FOXM1 is well known for its resistance development against many chemotherapies especially cisplatin. Thus, patients with progression of Class 2 tumors may not benefit from platinum-based chemotherapies. Patients with high FOXM1 expression seemed to profit most from BCG instillation therapy. However, only 8 patients of 346 patients from the low-risk FOXM1 group experienced progression, limiting the statistical power for the low-risk group. Thus, further studies for investigating the impact of BCG instillation therapy on patients with low FOXM1 expression are required.

In recent data, FOXM1 has also been shown to be an independent predictor for OS and disease-specific survival in muscle-invasive bladder cancer, with a subtype-specific expression in the luminal subtype (Rinaldetti et al. 2017). Thus FOXM1 has global impact on bladder cancer given its role in both muscle invasive and non-muscle invasive tumors. Up- and downstream FOXM1 regulators (e.g., FOXO3, PI3k, AKT) may be valuable drug targets and should be further explored also in bladder tumors (Yao et al. 2017).

This study has an exploratory character. FOXM1 needs to be further validated in prospective clinical studies in order to evaluate its impact in MIBC resistance development against platinum-based chemotherapies and to validate its prognostic role in NMIBC BCG instillation treatment. In this study two different quantification platforms (RNA-Seq vs microarray) allowed a distinct FOXM1 risk stratification. In order to translate these findings into clinics, a standardizable FOXM1 qPCR screening is needed for future studies (Rinaldetti et al. 2017).

The impact on MIBC and NMIBC as prognostic biomarker superior to clinicopathologic parameters and MKI67, raises FOXM1 to a crucial biomarker for molecular grading and to valuable drug target in bladder cancer (Radhakrishnan and Gartel 2008).

## Conclusions

FOXM1 is a highly prognostic marker for bladder cancer disease progression. It is mainly expressed in Class 2 and genomically unstable molecular bladder cancer subtype. As FOXM1 is a druggable proto-oncogene, the elucidation of its impact on bladder cancer survival may contribute to a further personalization of future NMIBC or MIBC therapy.
